# Mesoporous Silica Nanoparticles as a Potential Nanoplatform: Therapeutic Applications and Considerations

**DOI:** 10.3390/ijms24076349

**Published:** 2023-03-28

**Authors:** Krismala Djayanti, Pooja Maharjan, Kwan Hyung Cho, Sehoon Jeong, Man Su Kim, Meong Cheol Shin, Kyoung Ah Min

**Affiliations:** 1 College of Pharmacy and Inje Institute of Pharmaceutical Sciences and Research, Inje University, 197 Injero, Gimhae 50834, Republic of Korea; 2 Drug Delivery Disposition and Dynamics-Monash Institute of Pharmaceutical Sciences, Monash University (Parkville Campus), 381 Royal Parade, Parkville, VIC 3052, Australia; 3 Department of Healthcare Information Technology, Inje University, Gimhae 50834, Republic of Korea; 4 Institute for Digital Antiaging and Healthcare, Inje University, Gimhae 50834, Republic of Korea; 5 College of Pharmacy and Research Institute of Pharmaceutical Sciences, Gyeongsang National University, 501 Jinju Daero, Jinju 52828, Republic of Korea

**Keywords:** silica nanoparticles, nanoplatform, functionalization, drug carrier, biomedical application, cancer, tissue engineering

## Abstract

With advances in nanotechnology, nanoparticles have come to be regarded as carriers of therapeutic agents and have been widely studied to overcome various diseases in the biomedical field. Among these particles, mesoporous silica nanoparticles (MSNs) have been investigated as potential nanocarriers to deliver drug molecules to various target sites in the body. This review introduces the physicochemical properties of MSNs and synthesis procedures of MSN-based nanoplatforms. Moreover, we focus on updating biomedical applications of MSNs as a carrier of therapeutic or diagnostic cargo and review clinical trials using silica-nanoparticle-based systems. Herein, on the one hand, we pay attention to the pharmaceutical advantages of MSNs, including nanometer particle size, high surface area, and porous structures, thus enabling efficient delivery of high drug-loading content. On the other hand, we look through biosafety and toxicity issues associated with MSN-based platforms. Based on many reports so far, MSNs have been widely applied to construct tissue engineering platforms as well as treat various diseases, including cancer, by surface functionalization or incorporation of stimuli-responsive components. However, even with the advantageous aspects that MSNs possess, there are still considerations, such as optimizing physicochemical properties or dosage regimens, regarding use of MSNs in clinics. Progress in synthesis procedures and scale-up production as well as a thorough investigation into the biosafety of MSNs would enable design of innovative and safe MSN-based platforms in biomedical fields.

## 1. Introduction

Drug delivery is an enthralling field of research that has bagged the interest of researchers as delivering medicine to its target site of therapeutic action at a controlled rate is one of the core limitations of pharmaceutical industries. With ever-evolving approaches to targeted drug delivery, nanotechnology—a hot topic that has thus been conceptualized and invented—has modernized the drug delivery system and changed pharmaceutical approaches to circumvent a wide range of complications allied with drug delivery [1,2].

Recently, researchers have been actively investigating application of nanotechnology in biomedicine for diagnostic purposes, disease monitoring, and controlling degenerative diseases [3,4,5]. By applying nanotechnology, different types of nanoparticles have been created [6,7,8,9]. These nanoparticles have attracted many pharmaceutical and biomedical researchers as they can be modified for providing targeted drug delivery or controlled drug release from a single dose with improved bioavailability, thereby reducing toxicity. This makes them potential platforms for simultaneous therapy and biomedical imaging diagnosis [5,10]. Accordingly, they are usually referred to as nanotheranostics.

To date, various types of carriers (i.e., organic or inorganic materials) have been studied to overcome the major constraints and serve as a possible drug delivery vehicle to treat some particular diseases [11,12,13]. Their comparable sizes to biomolecules, biocompatible nature, and tissue specificity make them ideal functional probes for theranostic applications. Moreover, the recent development of surface-functionalized and architectural inorganic-nanomaterial-based drug delivery vehicles have become the center of attention for availing novel research opportunities in this burgeoning arena of biotechnological and biomedical applications and research [14,15]. Amongst various inorganic nanomaterials, mesoporous silica nanoparticles (MSNs)—which are among the key innovation precedents in material sciences—have become remarkable nanoplatforms and gained the attention of many researchers for cancer imaging and its therapy [16,17,18].

**Figure 1:**
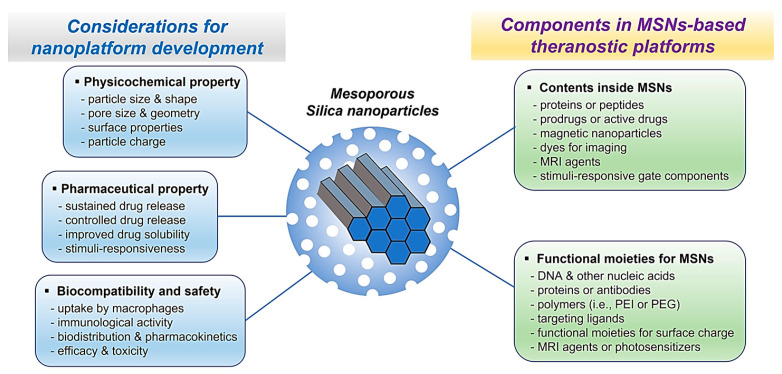
Mesoporous silica nanoparticle platforms used for drug delivery system: considerations for MSNs-based drug development process and components for MSNs-based theranostic system.

As one of the most useful nanoplatforms, MSNs are being immensely used for targeted or controlled drug delivery in cancer imaging and therapy as they have the potential to become useful therapeutic and diagnostic–theranostic tools [16] (Figure 1). MSNs have emerged as a promising vehicle for drugs or genes due to their tunable size and porous structure, preserving physicochemical stability and surface functionality, all of which ensure targeted or controlled delivery of various drug molecules. Therefore, MSNs can be used in a variety of salient features as nanosensors, nanomarkers, and drug delivery nanoplatform applications [19,20,21]. MSN-based multifunctional nanocomposites provide unique opportunities for simultaneous diagnosis and therapy and also serve as an imaging modality. In addition, due to the better biocompatibility, lower toxicity, increased surface area with small particle sizes, as well as uniformly sized pores with high pore volume enabling higher drug-loading content and controlled drug release, MSNs are being widely used as a vehicle in controlled and targeted drug (or gene) delivery systems or bioimaging agents (i.e., biosensors) [22,23].

With use of MSNs, premature release or degradation of drugs inside the MSNs pores and the deactivation effects of those drugs before reaching the target site can be prevented, which enables controlled drug delivery; in addition, MSNs can be used as luminescent sensors or photocatalysts in photodynamic treatment [5,24]. Considering the broad function of MSNs, they have been extensively used in biomedical applications, such as bone/tendon tissue engineering, diabetes, or inflammation [25]. However, most of the recent studies have focused on MSNs as nanocarriers for anti-cancer therapeutics. While most chemotherapeutic drugs, such as paclitaxel, docetaxel, doxorubicin, and so forth, effectively kill cancer cells, they also carry toxicity to normal healthy tissues [26,27]; therefore, using MSNs as a carrier for chemotherapeutic drugs can overcome the toxicity of conventional anti-cancer agents.

Considering the unique properties of MSNs, in recent years, theranostic application of MSNs has received much scholarly attention. Numerous previous studies have been performed to demonstrate effectiveness of MSNs as drug delivery systems along with detection of selective diseases. In this review, we overview the pharmaceutical properties of MSNs as drug delivery systems and the synthesis procedures for developing those particle-based platforms. This review also provides a highlighted role of MSNs in pharmaceutical applications to treat cancer and other diseases, including degenerative diseases, metabolic diseases, and infectious diseases. Aside from DDS based on MSNs, tissue engineering using MSNs is also discussed as an important strategy to improve degenerative diseases. We discuss the advantageous aspects and challenging issues of MSNs-based DDS for drug delivery to target diseases. Furthermore, we look through clinical studies using silica nanoparticles and comment on the safety issues of MSNs for biomedical use.

## 2. Physicochemical Properties of Mesoporous Silica Nanoparticles

MSNs consist of a honeycomb-like porous structure with a uniform pore size that enables encapsulation of large amounts of drug cargoes [28,29]. Their advantageous features include tunable pore size with a narrow distribution, internal mesoporous structures with huge pore volume and ordered arrangement, large surface area with abundant surface silanol groups, good chemical and thermal stability, as well as robustness and easy surface modification, making them potentially suitable to design multifunctional nanosystems [28,30]. The unique mesoporous structure and active surface present on MSNs enable surface functionalization and attachment of different functional groups, thereby enabling targeted and controlled delivery of therapeutic agents to a particular site in the body [16,23]; accordingly, MSNs have been extensively explored in different fields for theranostics, including biosensing, separation, or enzyme catalysis [18,22,31,32]. Furthermore, MSNs could be actively internalized into cells due to the surface silanol groups possessing affinity to phospholipids of cell membranes [33,34]. Due to the property of a high surface-to-volume ratio, MSNs have advantages as a better agent for cargo-loading purposes.

More specifically, the unique and expedient properties of MSNs can be summarized as follows [16,30,35]: (1) porous structure with uniform pore size and large pore volume; (2) large surface area and tunable size of particles; (3) functionalization of internal/external surfaces; (4) biocompatible and biodegradable particles. MSNs have a very narrow pore size distribution, with tunable pore diameter between 2 and 6 nm, so different drugs can be loaded and drug release profiles can be reliably investigated [34]. The large surface area (>900 m^2^/g) and pore volume (>0.9 cm^3^/g) enable high drug loadings [36]. MSNs with particle sizes between 50 and 300 nm can experience cellular uptake via endocytosis without causing significant cytotoxicity [37]. Compared to other polymer-based vehicles, MSNs are more resistant to mechanical stress, pH change, heat, or hydrolytic degradation process [38]. Generally, MSNs consist of a high density of silanol groups that can be altered using a broad range of organic moieties. MSNs have unique surface structures, including two functional surfaces, such as a surface of inner cylindrical pores and an outer surface of particles, which promotes functionalization of the inner or outer surfaces of MSN with different types of moieties [39]. The surface charge of MSNs can be controlled and chemically coupled with various functional compounds or molecules in internal and external pores. Furthermore, these surface-functional groups can also help in controlling the entrance pore size for entrapping molecules into the nanopores. Moreover, MSNs modified with surface functionalization minimize particle aggregation and escalate stability and redispersion. Without these physicochemical properties, conventional drug delivery systems have various limitations, such as physicochemical instability, particle aggregations, or poor aqueous dispersion [17]. Without functionalized surface chemistry, particle systems tend to possess less specificity, resulting in delivering drugs to targeted cells and normal healthy cells. Side effects or toxicity issues have been reported with DDS using nanoparticles, and size ranges, surface charges, or shapes of particles are known as important factors determining nanotoxicity [23]. Most importantly, after delivering drugs, the particle vehicles should either be excreted within several hours from the body or biodegraded inside the body into nontoxic products (i.e., silicic acid) [17]. Severe toxic effects often occur when using particles that are less biodegradable or biocompatible [13]. Biocompatible particles such as MSNs or liposomes do not assemble in the human body, and it has been reported that these non-viral vehicles do not cause severe biological effects during administration of longer-term treatment compared with viral vectors [4,23]. Owing to the inevitable physicochemical properties, many previous studies investigated MSNs in biomedical applications, specifically using theranostic approaches. However, according to several reports, the interaction between particles and specific cell types (i.e., red blood cells or fibroblast cells) can be influenced by the sizes, shapes, or surface chemistry of MSNs [40]. To guarantee drug efficacy and biosafety, the physicochemical properties of MSNs should be determined in further in vitro and in vivo experiments. In this review, we attempt to examine the toxicity issues associated with silica nanoparticles with several examples of research after discussing the therapeutic applications of the particles.

## 3. Synthesis of Mesoporous Silica Nanoparticles

In the 1960s, synthesis of monodispersed silica materials was patented by various groups, resulting in creation of hollow fibrous and porous particles consisting of a crystallized phase when exposed to surfactants and low bulk density. In 1968, W. Stöber et al. introduced a sol–gel method as a synthesis process of monodispersed silica nanoparticles [41]. This method involves hydrolytic reaction of tetra-alkyl silicates in a homogeneous alcoholic solution with ammonia as a catalyst, which results in creation of ‘non-porous’ silica particles with a wide range of sizes—from several nanometers to some microns. The original sol–gel method introduced by W. Stöber et al. has been modified to prepare various types of MSNs [42,43,44]. Furthermore, numerous approaches, including sol–gel processing or templating, have been used for synthesis of MSNs, resulting in a variety of engineered particle and pore sizes [37].

### 3.1. Sol–Gel Processing of Mesoporous Silica Nanoparticles

**Figure 2:**
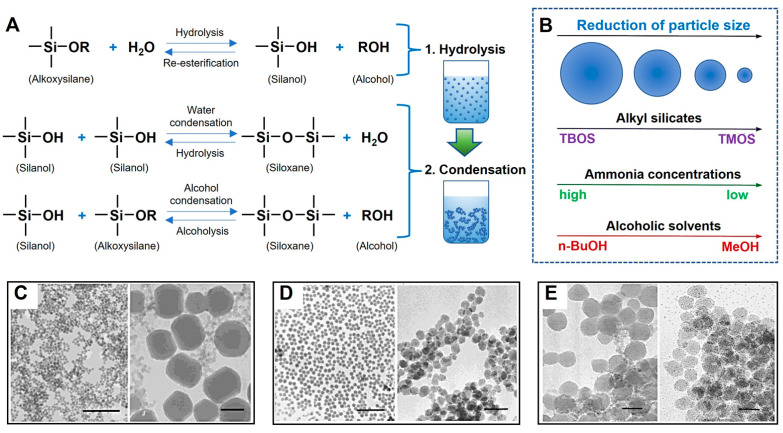
Synthesis of MSNs via hydrolysis and condensation process of sol–gel method [45]. (**A**) Scheme of a sol–gel method for production of MSNs. (**B**) Diagram showing the effect of reaction parameters on the size of MSNs in the synthesis process (reprinted with permission from V. Valtchev et al. (2013) [47]. Copyright 2013 American Chemical Society). (**C**–**E**) TEM images of MSNs produced by the sol–gel process (reproduction with permission from C.E. Fowler et al. (2001) [48]. Copyright 2001 John Wiley and Sons). (**C**) Images of MSNs prepared by a sol–gel process with a 220 s delay between the steps of dilution and neutralization. (**D**) Images of MSNs prepared by the sol–gel process with a 60 s delay between the steps of dilution and neutralization. (**E**) TEM images of DNP-functionalized MSNs.

The simple, low-cost process of the sol–gel method has been used to produce MSNs with unique surfaces and mesoporous structures (Figure 2). This process consists of the following two stages: hydrolysis and condensation reactions [45]. Hydrolytic reactions produce colloidal particle solution that can be stimulated in a wide pH range, acidic or alkaline pH. By contrast, condensation reaction occurs at neutral pH, resulting in gel-like formulation with 3D networks by crosslinking reaction in sols with siloxane bonds. After the particles undergo the drying process, various types of bioactive molecules could be embedded in silica gel matrix networks. Due to the porous properties and surface structures of MSNs, the final particle preparations can release the bioactive molecules in a controlled manner. This process can be used to prepare MSNs with a size range of 60–100 nm [37]. The advantages of this process are as follows: it is a simple 2-step process that is a time-saving and cost-effective process and can provide various types of MSNs possessing controlled mesopore structure and surface properties. S. Porrang et al. [46] reported that natural compounds from rice and wheat husks could be used to manufacture biogenic MSNs with a sol–gel process. They examined high anti-cancer activity against the MCF-7 cell line using biogenic MSNs loaded with doxorubicin.

### 3.2. Synthesis of Hollow Mesoporous Silica Nanoparticles by ‘Soft’ or ‘Hard’ Templating

**Figure 3:**
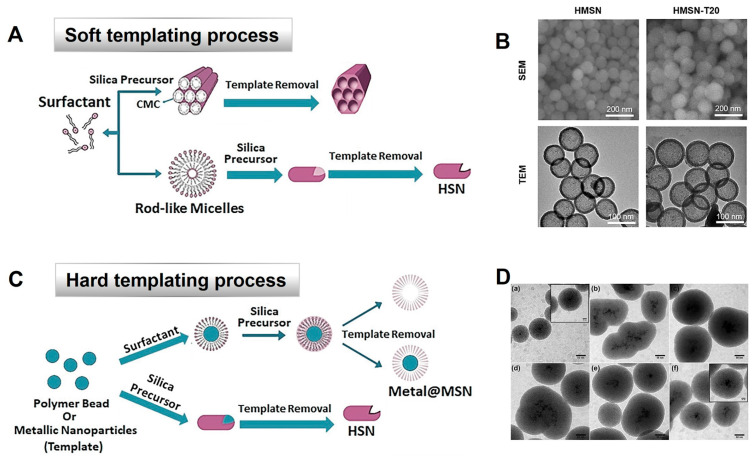
Synthesis process of MSNs by soft or hard templating. (**A**) Soft templating process and (**B**) TEM image of MSNs produced by soft templating (reproduction with permission from N.-H. Nguyen et al. (2022) [53]. Copyright 2022 Elsevier). (**C**) Hard templating process and (**D**) (**a**–**f**) TEM image of metal@MSNs intermediates produced by hard templating using different conditions (i.e., pH, amount of tetraethyl orthosilicate (TEOS), and ratio of ethanol-to-water) (reproduction with permission from O.A. Saputra et al. (2022) [52]. Copyright 2022 Elsevier). Templating diagrams (**A**,**C**) were reproduced with permission from F. Farjadian et al. (2019) [17]. Copyright 2019 Elsevier.

Hollow-type MSNs with mesoporous structures in the hollow space have increased pore volume and high internal/external surface area, all of which make them promising vehicles for drug delivery with improved drug loading capacity. Hollow silica particles could be prepared by forming vesicular-type structures, i.e., microemulsion or micelles as soft templates [49,50]. As mentioned above, the soft templating process is a simple method for preparing hollow-type MSNs. In this process, surfactants are used as templates or co-templates, and soft materials, such as vesicles, microemulsion, or micelles, are used as soft templates. The preparation process of soft-template MSNs is simpler than that of hard-template MSNs, and MSNs are produced under mild conditions with the soft templating method. Accordingly, amphiphilic molecules with both hydrophilic and hydrophobic domains are used as templates to direct synthesis, while the silica coating is susceptible to deformation during the coating procedure. Meanwhile, the soft templating process has limitations, such as the difficulty of complete removal of the template while preserving the nanoparticles with good dispersibility. Although the soft templating method has been widely used for preparation of MSNs, the broad size distribution of particles and co-existence of mixed mesostructures remain as important challenges in the formulation process. To overcome these issues by soft templating, hard templating materials, such as silica colloids, polymer lattices, or metal oxides, have been used to prepare MSN formulations with monodispersity [51]. The hard template maintains its shape with its rigid structure, which can be made from polymer, organic material, biological, or metallic components. Moreover, this method provides a more accurate structure and a wider range of sizes and ensures uniformity of product. Mesoporous materials or solid nanocrystal forms can be produced by hard templates, with a pore wall scale ranging from 2 to 50 nm [17]. Figure 3 represents the synthesis process of MSNs by soft or hard templating. O.A. Saputra et al. [52] produced hollow mesoporous silica with curcumin using the hard templating method, demonstrating a successful result, including an increase in curcumin loading onto the particles. Synthesis of a hard template made by sonically assisted co-precipitation with adding of L-serine was confirmed by XRD and TEM characterization.

## 4. Multifunctional MSNs for Theranostics

Recently, the theranostic (i.e., therapeutics and diagnostics) approach has become a matter of interest for many researchers in biomedical sciences; therefore, different studies explored drug delivery systems carrying agents with therapeutic action and having a significant effect in medical imaging and diagnosis [27]. Moreover, multifunctional theranostic nanoplatforms have been developed by incorporating both therapeutic and diagnostic potentials into a selected biocompatible and biodegradable nanoparticle to manage specific diseases. Basically, theranostic nanoparticles should be safe to use and rapidly reach the selected target site without affecting healthy tissues or any organs; in addition, they are also expected to efficiently release the required amount of active drug. With these new strategies, researchers have studied nano-inorganic systems as vehicles for bioactive molecules for theranostic purposes.

Amongst many nanoinorganic particles, such as titanium dioxide, silver, zinc oxide, gold nanoparticles, copper, and so forth, MSNs have been considered as one of the effective drug delivery systems with theranostic properties [18,23]. Accordingly, MSNs have been extensively applied in bone tissue engineering, diabetes mellitus, inflammation, and cancer, as well as in imaging and detection. Due to topologically distinct regions, i.e., hexagonal nanochannels/pores, silica framework, and particle exterior, which makes them independently functionalized, these MSNs are suitable for theranostic application [32,39,54]. Functionalization can be carried out by surface modification and integration of various bioconjugated moieties for particular diagnosis and therapy [14,26]. Since MSNs have been used in biomedical applications for various purposes, in the present review, we overview development of a variety of modified functionalized MSNs in terms of the therapeutic approach in cancer and various diseases. By adding multiple functions to the nanoparticles, multifunctional nano-engineered drug delivery systems with a combination of two or more compounds have been widely studied to improve therapeutic activity and minimize unacceptable side effects of noxious drugs [55,56]. Overall, these multifunctional nanoparticles are potential nanoplatforms for biomedical imaging diagnosis and therapeutics, i.e., theranostics.

Due to their astounding potential as nanoplatforms for diagnosis and treatment, MSNs have attracted much scholarly attention. Since MSNs possess better biocompatibility, huge surface area, and mesoporous structures with uniform pore size distribution and elevated pore volume, which permits further chemical modification, various studies have been carried out on using multifunctional MSNs as sustainable drug carriers [34,57,58,59]. In general, MSNs have been used to construct representative drug delivery systems (DDS), such as sustained DDS and stimuli-responsive controlled DDS. In the sustained DDS based on MSNs, incorporated drugs are released via a diffusion process (concentration gradient) from ordered mesoporous channels towards the media, and this diffusion process rapidly occurs once the nanocarriers get in contact with the media; thus, release of the incorporated drug in this delivery system has no temporal control [60,61]. By contrast, stimuli-responsive controlled DDS using MSNs are designed by employing efficient adsorption of organic molecules, such as fluorophores for imaging, hydrophilic polymers, gatekeepers, and targeting ligands on the surface of MSNs, that use internally or externally applied stimuli producing either physical or chemical responses, resulting in releasing incorporated drug molecules as per need [62,63]. Accordingly, in recent years, these physical and chemical surface modifications have expanded the range of theranostic applications for MSNs [64,65,66]. Overall, such progresses in surface modifications contributed to increasing biocompatibility and inhibition of specific adsorption and enabled functional groups in biomolecule conjugation.

## 5. Therapeutic Application of MSNs

### 5.1. Application of MSNs in Cancer Therapy

According to the announcement by Global Cancer Statistics (GLOBOCAN) and the World Health Organization (WHO), in 2020 alone, 19.3 million cases and almost 10 million deaths from cancer were reported in 185 countries [67]. Amongst 36 various cancer types, lung carcinoma is the predominant one that is responsible for an 18.4% mortality rate, followed by other cancers, such as colorectal, liver, stomach, cancer, and female breast cancer [67]. Cancer causes uncontrollable proliferation of abnormal cells that can lead to rapid neoangiogenesis, allowing only smaller particles to be preferentially transported and accumulate in the tumor, with enhanced permeability and retention effect. To date, therapeutic approaches, such as surgical removal, radiotherapy, and chemotherapy, have been applied for cancer treatment. However, these approaches have various constraints, such as poor aqueous solubility, rapid degradation, as well as low bioavailability and specificity [68]. Therefore, in the last several decades, many studies have been carried out on nanoparticles and their potential use in diagnostic and therapeutic applications in carcinoma treatment; some of these studies reported promising results [69,70]. Therefore, use of nanotechnology in cancer diagnosis and therapy is expected to provide hope to millions of cancer patients by providing a safer and more affordable treatment with limited toxic side effects. To date, MSNs as nanocarriers have been reported to have a significant effect on anti-cancer therapeutics delivery. Basically, multifunctional MSNs can target specific tumor sites, which increases the effective concentration of a particular drug to reach out to the specific site, with no harm to other healthy cells in the environment.

However, the size range of particles plays a crucial role in how bioactive drugs reach the target organ. For instance, particle size with a diameter exceeding 200 nm can be readily entrapped in the liver, spleen, and lung capillaries; furthermore, particles with a diameter below 200 nm can eliminate via structured tumor vasculature and then escape from hepatic and splenic filtration [71]. Particles with a size above 150 nm become trapped inside the liver and spleen, while particles with a diameter of less than 5 nm can be filtered out through the renal system [71]. All these aspects have to be considered while developing nanoparticles for cancer theranostics, such as in development of hybrid nanoparticles with multifunctional characteristics. Owing to the importance of nanoplatforms, several organic particles, such as liposomes, polymers, and inorganic particles, have been developed for cancer treatment [68,69]. Among these various nanoparticles, silica particles were usually believed to be safe and proposed as drug delivery vehicles in early 1983 [72]. Since then, a variety of silica materials in the form of drug delivery matrixes have been proposed. Several previous studies have used various functionalized MSNs. Types of these MSNs are discussed below.

#### 5.1.1. Surface-Functionalized MSNs in Cancer Therapy

In recent years, many studies have investigated various types of surface-functionalized MSNs for cancer therapy. For instance, in 2019, M.Y. Hanafi-Bojd et al. [65] developed functionally modified MSNs with polyethyleneimine–polyethylene glycol (PEI–PEG) or polyethylene glycol groups for carrying epirubicin hydrochloride against colorectal cancer cells, which resulted to be non-toxic to normal cells and were found to be significantly effective in inhibition of tumor growth cells from in vivo tumor suppression.

**Figure 4:**
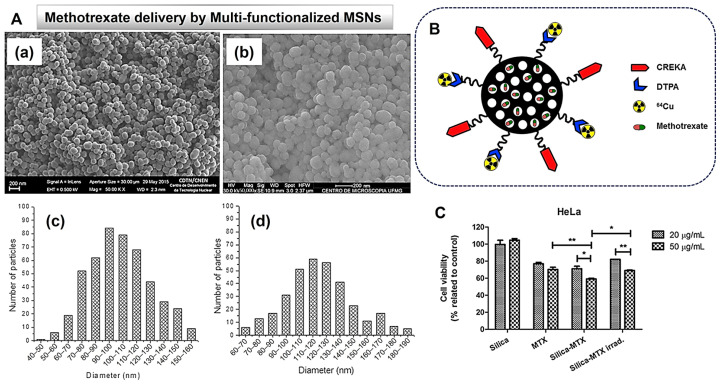
An example of multi-functionalized MSNs for cancer targeting, (**A**) SEM images of MSNs; (**a**), MCM-41, (**b**) final formulation as ‘MCM-41-APTES-mal-DTPA-CREKA-Cu’, particle size analysis by DLS (dynamic light scattering); (**c**) MCM-41, (**d**) MCM-41-APTES-mal-DTPA-CREKA-Cu. (**B**) Schematic diagram of MSNs formulation of ‘MCM-41-APTES-mal-DTPA-CREKA-Cu’ to deliver methotrexate to cancer cells. (**C**) Cell viability results with free MTX or MTX-loaded MSNs at different drug concentrations in HeLa cells. * *p* < 0.05; ** *p* < 0.01. Reproduced with permission from L.B. de Oliveira Freitas et al. (2017) [16]. Copyright 2017 Elsevier.

Furthermore, Z. Wang et al. [73] have experimented with use of surface-functionalized MSNs for imaging cancer cells (Figure 4). Specifically, the research group developed folic-acid-functionalized fluorescent MSNs consisting of 9,10-distyrylanthracene fluorogen core and folate-functionalized silica shell, which then were used in bioimaging of human cervical carcinoma (HeLa) cells and mouse 3T3-L1 preadipocytes. The results showed that the MSNs carried the property to target cancer cells overexpressing the folic acid receptors and avoid the normal cells, which do not overexpress such receptors. These results suggest a potential nanomaterial for cancer theranostic purposes. Similarly, a study carried out by N.-T. Chen et al. [74] has strongly suggested use of surface-functionalized MSNs for premalignant diagnosis of colonic lesions. The research group designed fluorescent/PEGylated MSNs labeled with Ulex Europaeus Agglutinin-1 (UEA1) to target premalignant lesions, resulting in early-stage colorectal polyps detection with higher sensitivity than that of the conventional colonoscopy methods.

#### 5.1.2. Stimuli-Responsive DDS Using MSNs in Cancer Therapy

Drug delivery systems for cancer therapy can be developed to enable controlled drug release in the targeted sites in the body by responding to stimuli in the biological system. An ideal stimuli-responsive nano-delivery system should be able to select and target a specific tumor microenvironment with high sensitivity and specificity; in addition, it should also be responsible for precise release of active drugs in response to internal/external stimuli. To date, stimuli-responsive controlled DDS based on MSNs have been designed using stimuli such as pH, redox state, temperature, enzymes, light, magnetic field, or any combination of the aforementioned stimuli [75]. Stimuli signals can be either endogenous or exogenous. Endogenous stimuli generally occur due to differences in pH value, redox state, and biomolecules between the intracellular environment of neoplastic and normal tissues.

Various studies have reported enhanced therapeutic efficacy by employing stimuli-responsive controlled DDS based on MSNs with no leakage of incorporated bioactive therapeutic agents and increased transportation of those molecules in the specific target tumor site. Therefore, numerous previous studies have investigated stimuli-responsive modification of nanosystems specifically with MSNs for carcinoma therapy. As discussed previously, unique properties of MSNs include their exceptionally large surface area, uniform pore size distribution, and pore volume that leads to increased lodgment of drug molecules in higher concentrations within pore channels. Furthermore, such modification facilitates linkage of various gatekeepers on the pore entrance, leading to controlled and sustained drug release from the channels to the target site while limiting side effects by delivering drugs at high concentrations to healthy and normal tissues. Moreover, with such a modification using the stimuli-responsive delivery system, the antitumor drugs can be protected from unwanted degradation in harsh and extreme surroundings, i.e., the acidic environment in the stomach. Especially, MSNs considered to be rigid building blocks for accommodating drug compounds have been reported to prevent premature release of loaded drugs and also enable controlled release of the drug once the target tumor is reached. The mechanisms of some of the stimuli are summarized in Table 1.

In recent years, various studies have been carried out regarding stimuli-responsive MSNs for different types of cancer theranostics. For instance, L. Yang et al. [83] developed MSNs loaded with anti-microRNA-155 against an oncogenic microRNA upregulated in human colorectal carcinoma and modified MSNs-based formulation with polymerized dopamine and AS1411 aptamer for colorectal cancer therapy. In this modification, polymerized dopamine is a pH-responsive gatekeeper that promotes controlled drug release in the acidic microenvironment of malignant cells, thereby providing these functionalized MSNs with significantly higher efficiency in targeting tumors and better therapeutic efficacy in both in vivo and in vitro studies and making them promising carriers for colorectal carcinoma therapy. Meanwhile, M. Martinez-Carmona et al. [84] developed novel multifunctional MSNs loaded with doxorubicin (DOX), which have been layered externally by polyacrylic acid (PAA) and could deliver drugs by responding to the pH of the external environment; these MSNs were modified with targeting ligand, i.e., plant lectin concanavalin that targets overexpressed sialic acids, surface glycans, in tumor cells. The research group reported a higher degree of internalization of these multifunctional MSNs into human osteosarcoma cells as there was overexpression of sialic acids, and they further promoted pH-responsive controlled release of the low concentration of loaded DOX (2.5 µg/mL), resulting in death of nearly 100% of osteosarcoma cells without affecting healthy osteocytes in vitro. Together, the results of this study suggest that these multifunctional stimuli-responsive MSNs are promising nanoplatforms for treatment of bone cancers.

**Figure 5:**
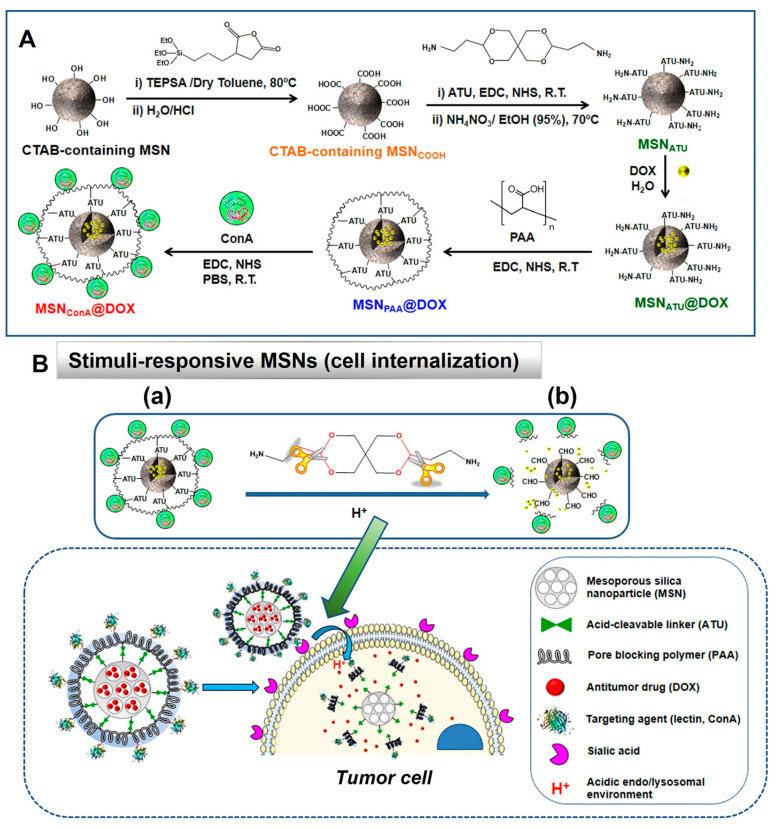
Fabrication of lectin-conjugated pH-responsive MSNs for cancer therapy as an example of the stimuli-responsive system. (**A**) Synthesis process of doxorubicin (DOX)-loaded MSNs. Particles were prepared by grafting with a pH-cleavable linker (ATU: 3,9-Bis(3-aminopropyl)-2,4,8,10-tetraoxaspiro [5,5] undecane) and coating with an acid-degradable polymer (PAA: poly(acrylic acid)). (**B**) Schematic diagram of cell internalization of pH-responsive MSNs and drug release in the targeted cells. After the cell internalization via interaction between ConA (targeting agent) of MSNs and cell-surface glycans overexpressed on tumors, the constructs of MSNs can change from the structure (**a**) into (**b**) by the cleavage of pH-responsive linker in endo/lysosomes with acidic pH in the cells, resulting in drug release to eradicate the tumor cells. Reproduced with permission from M. Martínez-Carmona et al. (2018) [84]. Copyright 2018 Elsevier.

Furthermore, D. Zhu et al. [85] designed novel MSNs by applying a dual-sensitive (enzyme and redox) mechanism by functionalizing MSNs polymerized with cystine-dopamine and loaded with DOX; the produced MSNs proved to be responsive to glutathione (GSH) and pepsin with controlled drug release (Figure 5). They also proved to be non-toxic, highly biocompatible, and suitable for use as drug nanocarriers in controlled DDS for sustainable cancer therapy.

On the other hand, stimuli-responsive MSNs showed potential to be used as nanodevices for theranostic purposes in cancer diseases. For instance, H. He et al. [63] developed GSH-responsive MSNs for cancer therapy and mitochondrial targeted imaging by combining MSNs with gold nanoparticles (as gatekeepers) along with cationic ligand (triphenylphosphine) and loaded DOX onto it; carbon nanodots were used for fabrication of mitochondrial targeting drug delivery system. This novel formulation released the loaded DOX into cells in a GSH-dependent manner. Furthermore, this nanodevice can serve as a potential fluorescent probe for mitochondria-targeted fluorescent imaging in living cells, therefore holding great potential theranostic approaches. Furthermore, H. Lu et al. [86] created a multiple stimuli-responsive nanodelivery system by novel drug formulation, i.e., mesoporous-silica-coated carbon nanocomposite loaded with DOX. Since carbon dots were covalently attached to the surface of particles, the MSNs delivery system could serve as fluorescent probes for imaging and also as photothermal therapeutic agents for cancer treatment. As a result, in vitro drug release proved that the mesoporous-silica-coated carbon nanocomposite was effective as a potential drug delivery vehicle, providing drug release in response to pH/redox/near-infrared stimuli; furthermore, the cytological and in vivo testing showed better chemo-photothermal synergistic anti-cancer treatment efficacy with the drug formulation. An experiment carried out by M. Sedighi et al. [87] successfully developed novel functionalized MSNs by capping them with inorganic cerium oxide nanoparticles as a gatekeeper that prevents premature drug leakage under physiological conditions, loaded with tyrosine kinase inhibitor such as sorafenib (SFN) and sunitinib (SUN) and applied to the hepatocarcinoma cells in vitro; the results showed better internalization of functionalized MSNs with intracellular release of SFN and SUN via induction of ROS production, which resulted in enhanced apoptotic cell death.

### 5.2. Application of MSNs for Other Diseases

Apart from cancer therapeutics, MSNs have been broadly utilized in treatment of various diseases (i.e., ocular diseases, bacterial infections, and so on). In the context of developing treatment of these diseases, various studies have been carried out by devising surface-modified MSNs or stimuli-responsive DDS based on MSNs.

#### 5.2.1. Surface-Functionalized MSNs in Other Diseases

Surface functionalization has been used to improve the properties of MSNs as vehicles. In one relevant study, Y.-T. Liao et al. [88] synthesized gelatin-functionalized MSNs for intracameral pharmacotherapy of glaucoma for maintenance of intraocular pressure and conducted in vivo studies on rabbit eyes. The results showed sustainable drug release from the functionalized MSNs with controlled intraocular pressure. Similarly, a study carried out by M.R.B. Paiva et al. [66] showed MSNs functionalized with 3-aminopropyl-triethoxysilane as promising ocular delivery for drug tacrolimus for treatment of eye diseases in anterior and posterior segments. Specifically, the research group observed an effective loading process of the drug; in addition, in vivo results showed that the produced MSNs were biocompatible and caused no side effects, such as neovascularization, vitreous hemorrhage, or abnormalities in the retina and optic nerve.

Furthermore, E.P.F. Nhavene et al. [89] used chitosan and succinic acid to functionalize MSNs for delivery of benznidazole for treatment of Chagas disease or American trypanosomiasis; the results proved efficient delivery of benznidazole to *T. cruzi* parasites by MSNs. With regard to osteoarthritis management, A. L. Mohamed et al. [90] developed cotton patches loaded with colchicine-containing MSNs/hydrogel composites and conducted ex vivo drug penetration through rat skin, resulting in higher drug penetration levels sustained for 24 h. The results also showed a protective effect against osteoarthritis via the patches, suggesting it is a potential formulation for management of osteoarthritis. S. Geng et al. [91] performed in vitro and in vivo studies to evaluate the N-EDMSNs/pFGF21/Lira complexes based on MSNs in treatment of type 2 diabetes mellitus (T2DM). They prepared the embedded dual MSN types (EDMSNs) and then modified the EDMSNs with amino functionalization (N-EDMSNs). Finally, fibroblast growth factor 21 (FGF-21) plasmid and liraglutide (Lira) were loaded on the N-EDMSNs. The complexes (N-EDMSNs/pFGF21/Lira) significantly inhibited gluconeogenesis in liver by inhibiting glucose-6-phosphatase and PEPCK activity and increased hepatic insulin sensitivity by inducing phosphorylation of insulin signaling molecules (InsR, IRS-1, P13K, and Akt) in the liver. This treatment significantly reduced food intake and body weight in the high-fat diet-fed mice along with lowering blood glucose levels, which means that the formulation could contribute to treating T2DM with obesity. In a recent study*,* J. Sun et al. [92] developed an anti-inflammatory nanoformulation for ocular vascular disease by using surface-modified (SM) hollow MSNs. The functionalized hollow MSNs (hMSN(SM)) were synthesized through -NH_2_ modification and then PEG-modification. MSNs were loaded with conbercept (CBC) (an anti-angiogenic macromolecule) and MCC950 (MCC, an anti-inflammatory drug) to prepare the complex formulation of CBC-MCC@hMSN(SM). This nanoformulation was found to have acceptable biocompatibility based on cytotoxicity tests and finally enhanced the anti-angiogenic and anti-inflammatory effects in retinal vasculature by potently suppressing diabetes-induced ocular vascular pathology.

#### 5.2.2. Stimuli-Responsive DDS Using MSNs in Other Diseases

**Figure 6:**
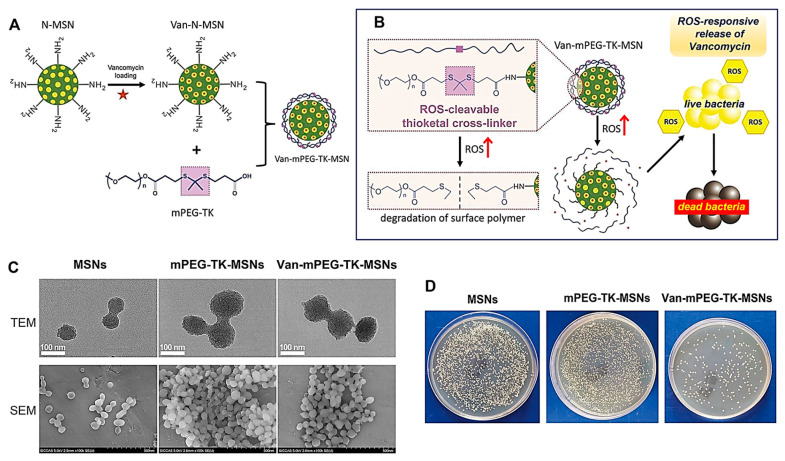
Fabrication of MSNs with ROS-responsive components as an antibacterial agent. (**A**) Scheme of synthesis procedure of vancomycin-loaded MSNs functionalized with TK (a ROS-responsive linker) and mPEG (methoxy poly(ethylene glycol)). (**B**) Diagram of ROS-responsive release of vancomycin from Van-mPEG-TK-MSNs in the infected site. (**C**) TEM and SEM images of each formulation type of MSNs. (**D**) Antibacterial activity results using S. aureus by each type of MSNs. Reproduced with permission from J. Li et al. (2020) [93]. Copyright 2020 Elsevier.

Microenvironmental stimuli can be varied under different disease conditions; thus, they have been utilized to construct MSNs-based DDS for improved drug efficiency or reduced toxicity. In J. Li et al.’s [93] study, stimuli-responsive MSNs were studied for treatment of bacterial infections (Figure 6). In this study, the stimulus was reactive oxygen species (ROS) released from live bacteria, and MSNs were modified with thioketal functionalized methoxy poly (ethylene glycol) as ROS responsive gatekeeper to deliver the antibacterial drug, i.e., vancomycin, in the infected sites. The results of the in vivo experiment showed that the infection recovered within 14 days, suggesting that ROS-responsive MSNs can be employed in advancement of novel antimicrobial agents. Z. Qu et al. [94] demonstrated that the pH-responsive formulation based on MSNs was highly efficient for targeted delivery of glucocorticoids to colon and reducing levels of inflammatory cytokines in murine models of inflammatory bowel disease. In the study, MSNs were loaded with prednisolone and budesonide and then coated with Eudragit S100, a pH-responsive polymer. In the in vitro dissolution test of uncoated vs. coated MSNs, burst release of drugs was limited in the case of Eudragit-coated MSNs, resulting in release of more than 60% drugs only at the colonic pH condition. Furthermore, in vivo experimental results supported that the pH-responsive MSNs could improve drug efficacy for inflammatory bowel disease by preventing premature drug release in other gastric regions.

Recently, MSNs have also been applied in therapy of neurodegenerative diseases including Alzheimer’s disease (AD) by using natural sources of curcumin that are believed to improve the therapeutic effect. Curcumin-loaded MSNs (MSN-CCM) with a combination of thermo-responsive hydrogel (HG) successfully reverted cognitive deficits in the streptozotocin-induced AD mouse model, as well as showed good mucoadhesive properties [95]. An ex vivo study reported that both MSN-CCM and HG@MSN-CCM have a high permeation rate in the porcine nasal mucosa; therefore, this research proved that the design formula can be a potential candidate for treatment of Alzheimer’s disease. Moreover, Y. Tao et al.’s [96] study gained attention on utilizing thrombus microenvironment to treat vein thrombosis, known as a serious type of cardiovascular diseases. They prepared a bowl-shaped nanomotor based on MSNs, functionally modified with arginine-glycine-aspartic acid (RGD) polypeptide, loaded with L-arginine (LA) and urokinase (UK) as thrombolytic drug. By reacting with excessive ROS in the thrombus site, LA in the MSNs produce nitric oxide, which can promote penetration of the MS/LA/RGD/UK nanomotor composites in the thrombus site. With enhanced retention efficiency of nanomotors in the sites of thrombosis, ROS levels were reduced and vascular injury caused by inflammation could be alleviated. Especially, this bowl-shaped MS/LA/RGD/UK formulation helped to stimulate growth and recovery of endothelial cells. Both in vitro and in vivo studies demonstrated the advantages of MSNs formulation for overcoming thrombosis with no significant toxicity.

### 5.3. Application of MSNs in Tissue Engineering

**Figure 7:**
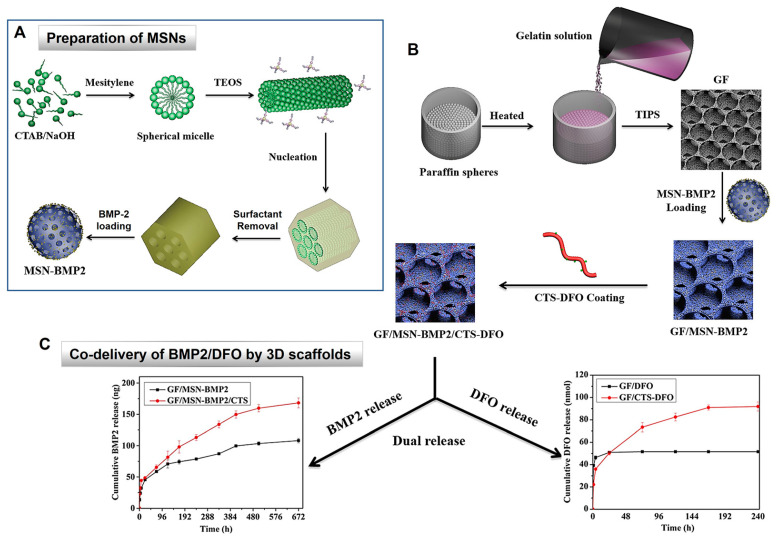
Application of MSNs/3D nanofibrous scaffolds for tissue engineering as a strategy of bone regeneration. (**A**) Synthesis of BMP2-encapsulated MSNs. (**B**) Preparation of 3D scaffolds of nanofibrous gelatin (GF) with MSN-BMP loading and CTS-DFO coating. (**C**) Co-delivery of BMP2 and deferoxamine (DFO) was enabled by a local and controlled release system based on MSNs and chitosan (CTS). Reproduced with permission from Q. Yao et al. (2018) [55]. Copyright 2018 Elsevier.

Thus far, MSNs have been broadly applied to carry various types of cargo, including small molecules to macromolecules, such as peptides, proteins, or genes. Table 2 shows MSN-based nanoplatforms used for treatment of cancers or other diseases [5]. Accordingly, several previous studies have investigated the potential of MSNs in the field of tissue engineering (Table 2). These studies showed several advantages of developing hybrid scaffolds incorporated with MSNs because of the aforementioned characteristics of MSNs (Figure 7). Moreover, the silicon (Si) ions from MSNs were found to have specific induction activities on stem cells upon release that help to accelerate the repair process. Apart from this, MSNs were also found to escalate the mechanical properties, i.e., the interaction between cells and materials of the MSNs-incorporated hybrid scaffolds.

To date, various research has been carried out on MSNs applications in tissue scaffolding. Q. Yao et al. [55] investigated a dual-drug delivery system with drugs, i.e., BMP2 and deferoxamine, for effective osteocyte regeneration using MSNs/3D nanofibrous scaffold. The results of this study demonstrated sustained release of BMP2 that maintained the osteogenic process following release of deferoxamine that enhances angiogenesis. Furthermore, MSNs have also been studied in cardiac stem cell regeneration. For instance, Y. Sun et al. [60] developed a three-dimensional mesoporous scaffold incorporated with insulin-like growth factor-1 (IGF-1). They demonstrated that the scaffolds supported proliferation of cells and sustainable/controlled release of IGF-1 from the scaffolds facilitated growth of cardiac cells.

In another relevant study, Y.-T. Hou et al. [97] devised a novel targeted drug delivery system with MSNs in which glycyrrhizin-conjugated, chitosan-coated, and lysine were embedded for accelerating hepatic functions, including hepatocyte regeneration. The results of this in vitro study showed that this drug formulation appeared to be a potential candidate. Similarly, there have been various studies in the area of skin tissue engineering. For example, S. Rathinavel et al. [61] designed SBA-15 mesoporous silica and fabricated it into PVA nanofiber and loaded it with curcumin for sustained release of the drug for wound healing. The results revealed that the synthesized nanoscaffold accelerated the wound healing process in rat models, therefore proving MSNs as one of the strong contenders for skin tissue regeneration.

Furthermore, in the domain of neural tissue engineering, applications of MSNs have widely been studied as well. For instance, J.H. Lee et al. [98] developed a cell culture system consisting of MSNs combined with collagen hydrogel for delivery of nerve growth factor (NGF); large proteins were loaded within the enlarged mesopores, resulting in significant up-regulation of growth-associated protein GAP43, which induced delivery of NGF. This helped in stimulation of neuritogenesis and maintained cell viability, ensuring the effectiveness of MSNs for brain tissue regeneration. With regard to axonal regeneration, M.S. Kim et al. [99] developed an MSN-based delivery system of bisperoxovanadium for inhibiting phosphatase and tensin homolog deleted on chromosome 10 (PTEN) in nerve system and experimented with the in vitro culture of the dorsal root ganglions. The results of this study showed the significant stimulation of neurite outgrowth and upregulation in the expression of signal molecules in association with the inhibition of PTEN along with maintaining cell viability. Furthermore, the research group tested the effects of the MSN-based preparations by directly injecting the formulation into nerve tissues, including the brain cortex, dorsal roots, and spinal cord. The results exhibited better synchronization of nanoparticles in the biological system, indicating the feasibility of MSNs in nerve cell engineering. Finally, recently, C.-S. Cheng et al. [56] studied the codelivery of curcumin and plasmid for treatment of neurodegenerative diseases using MSNs as a delivery platform. The research group used curcumin as an antioxidant for protection of cells against ROS-induced damage and plasmid RhoG-DsRed to elevate neurite outgrowth, and TAT peptide was combined to plasmid for a better delivery of plasmid in the cells that enhanced gene expression. Using N2a cells, the group reported that the neurite outgrowth was enhanced with gene expression and antioxidant activity of curcumin was observed.

**Table 2:** Overview of studies on MSNs-based biomedical applications, including cancer, other diseases, and tissue engineering [5].

Therapeutic Agents with Matrix or Scaffold	MSNs Properties				Examined Cell/ Animal Model	Target Disease	Research Outcomes	**Reference**
	Surface Area (m^2^/g)	Pore Diameter (nm)	Pore Volume (cm^3^/g)	Diameter (nm)				
HMSNs-CS-DOX@CuS	-	1.84	-	150 ± 13	MDA-MB-231 cells; Mice	Breast cancer	Both in vitro and in vivo showed excellent apoptosis effects on cancer cells and provided extended lifetimes in animal models, which can be a promising theranostic (PTT) method in cancer therapy.	Niu, 2021 [59]
CAP-MSN capped with CHS-GCA (CAP: capecitabine)	419.36 ± 6.98	8.12 ± 0.43	0.73 ± 0.21	245.24 ± 5.75	HCT 116 cells; Rats	Colorectal cancer	Drug was released in a controlled manner from (CAP-MSN)CHS-GCA for up to 72 h, providing a cytotoxic effect at low doses and reducing toxic effects on non-target organs.	Narayan, 2021 [62]
DOX@MSN-pTA (DOX: doxorubicin)	607	1.7	-	ca. 200	4T1 cells; Mice	Cancer	DOX@MSN-pTA displayed the highest cytotoxic effect in vitro and as a combined chemo-photothermal (PTT) therapy in vivo.	Shi, 2021 [100]
PTX@DMSN@PMAsh-Tf (PTX: paclitaxel)	376	13	1.36	ca. 100	A549 cells; Mice	Cancer	Particles were efficacious in inhibiting tumor growth in in vivo trials with high drug loading, good colloidal stability, and low cytotoxicity.	Deng, 2021 [101]
MTX-loaded MSN–APTES–chitosan (MTX: methotrexate)	789 ± 4	-	0.83	97.7 ± 8.8	MCF7 cells	Breast cancer	Breast cancer cell viability could be significantly affected by MTX-loaded MSN–APTES–chitosan at a relatively low dose	Shakeran, 2021 [102]
Curcumin-loaded MSN-HA-C	-	-	-	75–110	MCF-7 cells; MDA-MB 231 cells; Mice	Breast cancer	The nanohybrid design effectively reduced tumor volume and increased efficacy against cancer through induction of ROS, cell cycle arrest, and apoptosis rather than free curcumin.	Ghosh, 2021 [103]
Myr-loaded MSN (Myr: myricetin)				109.8	A594 cells; NCI-H1299 cells; Mice	Non-small-cell lung cancer (NSCLC)	The treatment of NSCLC by Myr-loaded MSN combined with MRP-1 siRNA can be effective given the fact that significant apoptosis occurs in cancer cells with the least amount of side effects.	Song 2020 [104]
DOX-loaded MSN@MPN	526.26	-	0.973	95–110	A549 cells	Lung cancer	Dox-loaded MSN coated with MPN provided a significant PTT effect and pH-triggered drug release to kill cancer cells effectively.	Yang, 2020 [105]
MNPSiO_2_-FA/Cis-Pt (FA: folic acid, Cis-Pt: cisplatin)	1011	3–5	1.10	100	LN18 cells	Glioblastoma cancer	The formulation showed a high cytotoxicity effect and excellent biocompatibility with the controlled drug release in a sustained manner.	Ortiz-Islas, 2021 [106]
NMS-MSNs-COOH; IMC-MSNs-COOH (NMS: nimesulide, IMC: indomethacin)	421; 392	1.8; 1.6	0.40; 0.38	232.5; 238.6	Rats	Inflammatory diseases	In vivo results demonstrated that NMS/IMC-loaded MSN-COOH could exert a strong anti-inflammatory effect by achieving higher bioavailability for NMS and IMC with increased dissolution.	Gou, 2021 [64]
MSN-A-Pre-Eu; MSN-A-Bud-Eu (Pre: prednisolone, Bud: budesonide)	26 ± 12; 20 ± 14	-	0.4 ± 0.02; 0.5 ± 0.02	238 ± 12.7; 242 ± 18.6	Mice	Inflammatory bowel disease	Compared to free drugs, the pH-responsive formulation based on MSNs reduced inflammation by preventing premature drug release and improving drug efficacy.	Qu, 2020 [94]
Ibuprofen	736.5 ± 15.29	2.4	-	150	Human Embryo Kidney cells	Inflammatory diseases	Using MSNs-based DDS, solubility, bioavailability, and stability issues of ibuprofen could be improved. Sustained drug release was enabled by MSNs, which reduces the frequency of dosing, thus reducing side effects related to NSAIDs.	Ortega, 2020 [107]
N-EDMSNs/pFGF21/Lira (Lira: liraglutide)	567	10.7	1.27	ca. 230	Hepa1–6 cells; Mice	Type 2 diabetes mellitus (T2DM)	MSNs-based DDS (delivering GLP-1AR (Lira) and FGF-21 plasmids) was more effective than non-MSN types in improving glucose tolerance and inhibiting PEPCK and G-6-Pase activity without causing toxicity or side effects.	Geng, 2021 [91]
VLG-SiNPs (VLG: vildagliptin)	962.5		0.95	310.9–383.68	-	Diabetes	A new DDS based on MSNs enabled sustained release of the antidiabetic drug, which might contribute to reducing the frequency of drug administration and ultimately enhancing patients’ compliance.	Shirsath, 2021 [108]
CBC-MCC@hMSN(SM) (CBC: conbercept, MCC: MCC950)	18.4	7.1	0.07	338.2	HRVECs; Mice	Ocular vascular disease	The formulation increased anti-angiogenic and anti-inflammatory efficacy, resulting in sustained suppression of inflammatory responses in the ocular tissues.	Sun, 2023 [92]
Van-mPEG-TK-MSNs (Van: vancomycin)	341.4	-	0.59	100	MC3T3-E1 cells; S. aureus; Rats	Bacterial infectious diseases	Van-mPEG-TK-MSNs exerted controlled release of antibacterial drug molecules via reactive oxygen species (ROS)-responsive delivery.	Li, 2020 [93]
SBA15@NH_2_/LVX/PLA-NF (LVX: levofloxacin)	163.96	5.4	0.011	-	HFB4 cells; *S. aureus*; *E. coli*; *C. albicans*; *A. niger*	Infectious diseases	MSNs-based formulation loaded with LVX improved antimicrobial efficacy and cytocompatibility, which could help to reduce side effects.	Abdelbar, 2020 [109]
HG@MSN-CCM (HG: hydrogel, CCM: curcumin-loaded mesoporous)	556	6.4	1.29	158.1 ± 9.64	L929 cells; Mice	Alzheimer’s disease (AD)	Animal groups treated with MSN-CCM or HG@MSN-CCM showed higher memory retention than those with CCM or HG@MSN. These MSN-based formulations regressed cognitive deficits in mice, suggesting their potential to treat AD.	Riberio, 2022 [95]
MSN-Ca-RV-PS (RV: rivastigmine)	36.20	4.20	0.25	>100	PC12 cells; Rats	Alzheimer’s disease (AD)	Brain uptake clearance, the plasma half-life of the drug, and the brain-to-plasma concentration ratio were improved by using MSNs compared with the free drug.	Basharzad, 2022 [110]
MS/LA/RGD/UK (LA: L-arginine, UK: urokinase)	192	-	0.6	255	HUVECs; Rats	Venous thrombosis	MSNs-based DDS reduced ROS levels, improved endothelialization processes with appropriate blood compatibility, and finally increased thrombolytic activity in vivo.	Tao, 2022 [96]
Q-MSNs (Q: quercetin)	-	-	-	100–150	Rats	Myocardial Ischemia-Reperfusion Injury	MSNs-based formula (Q-MSNs) helped to increase the pharmacological activity of quercetin by inhibiting cell apoptosis and oxidative stress and improving cardiac blood flow recovery.	Liu, 2021 [111]
MSN-NGR1-CD11b antibody (NGR1: notoginsenoside R1)	-	-	-	83	H9C2 cells; Mice	Myocardial infarction	The combined formulation of MSNs with NGR1 and CD11b antibodies enhanced drug delivery to the target site, showing increased cardiac function and reduced local inflammation in vivo. MSNs protected H9Cs cells from oxidative stress damage.	Li, 2022 [112]
SA-PSiO_2_-SeNDs-PEG (SA: synaptic acid)	-	-	-	188	HUVECs; Mice	Cardiovascular disease	The SA-PSiO2-SeNDs-PEG nanocomposite promoted more stable and sustainable drug release and no side effects. The formulation provided some health benefits, i.e., a reduction in ROS stress and a decrease in LDL-C level.	Bi, 2020 [113]
SIM@HA-MSN (SIM: simvastatin, HA: hyaluronic acid)	27.64	-	0.187	189.1 ± 5.8	Raw264.7 cells; HUVECs	Atherosclerosis	The results showed a new treatment for atherosclerosis with robust targeting, anti-inflammatory activity, and low toxicity by using MSNs. The formulation exhibited properties of lesion-targeting and long-circulation in blood.	Song, 2022 [114]
TNFR-Dex-MSNs (Dex: dexamethasone)	1167	2.5	0.96	222 ± 17	Mice	Acute lung injury	By reducing cytokine levels and side effects, MSNs-based DDS capped with a peptide targeting the TNFR1 receptor provided a more significant therapeutic effect than the free drug.	García-Fernández, 2021 [115]
Dex-loaded RMSNs	-	-	-	513.6 ± 63.1	Rats	Rheumatoid arthritis	Comparatively to the control group, Dex-loaded RMSNs showed significant anti-inflammatory effects and cartilage regeneration as well as high drug-loading efficiency.	Kim, 2022 [116]
CSL@HMSNs-Cs (CSL: celastrol; Cs: chitosan)	52.82	2.4	0.121	260.8–290.2	Chondrocytes; Rats	Knee osteoarthritis	A pH-responsive MSNs formulation loaded with CSL showed high solubility for intra-articular injection and good therapeutic effect for osteoarthritis by downregulating protein levels in the NF-κB signaling pathway in chondrocytes.	Jin, 2020 [117]
MSNs-PA@PEI; MSNs-PEG@PEI	-	2	-	150	MC3T3-E1 cells; Mice	Osteoporosis	As carriers of SOST siRNAs, MSNs showed superior results at increasing osteogenic expression and delivering active substances to the target site compared to PTH administration.	Mora-Raimundo, 2021 [118]
Ca-, Mg- and Sr- co-doped MSNs	668–1279	2.4–3.1	0.753–1.994	151.9–534.7	Human periodontal ligament fibroblasts (hPDLFs)	Tissue regeneration	A dope MSNs with Ca, Mg, and Sr was formulated for delivery of moxifloxacin and increased cell proliferation, hemolysis activity, and differentiation of osteoblasts in periodontal ligament cells for tissue regeneration.	Pouroutzidou, 2021 [119]
CHX-loaded/MSN-PGA (CHX: chlorhexidine)	-	-	-	84–98	*S. mutans*; Dental pulp stem cells (DPSCs)	Restorative dentistry	Having high antibacterial activity and penetration ability inside dentin and lowering MMP-8 and cathepsin K levels in dentin, the formulation was found to be suitable for adhesive and restorative dentistry.	Akram, 2021 [120]
PCL + Cur + SBA-15; PCL + Cur + NH_2_-SBA-15 (Cur: curcumin)	116.10	4.8	0.3	-	*B. Subtilis*; *E. coli*; Swiss 3T6 cells; Rats	Skin wounds	The nanofiber formulation based on SBA-15 or NH_2_-SBA-15 design exhibited high biocompatibility, cell adhesion, cell viability, antibacterial activity, and significant wound healing effects in vitro and in vivo.	Rathinavel, 2021 [57]
Ce@MSNs (Ce: ceria)	435.45 ± 10	3 ± 0.85	0.58 ± 0.01	>70 and <200	MC3T3-E1 cells; Raw264.7 cells	Osteoporosis	Ce@MSNs showed stable therapeutic effects and antioxidant activity, providing osteogenesis with low side effects.	Pinna, 2021 [121]
MSN@PEG/PEI–OGP (OGP: osteogenic growth peptide)	593	3.38	-	107.8 ± 1.0	MC3T3-E1 cells; Rabbits	Bone repair/regeneration	The MSNs-based formulation increased ALP activity and calcium deposition and also showed an excellent osteointegration effect for bone remodeling.	Chu, 2022 [122]
Alginate/Chitosan/MSN30	-	-	-	100	BMSCs cells	Implant for craniofacial bone defects	Compared to the control, treatment with Alginate/Chitosan/MSN30 had a significant effect on cell viability and a positive effect on osteogenesis.	Yousefasl, 2021 [123]
DMSNs/M-CAG (dexamethasone-loaded MSNs)	-	-	-	210.6	BMSCs cells; Rats	Bone tissue regeneration	A composite scaffold was shown to increase cell proliferation and stimulate osteogenesis in rats with calvaria bone defects.	Zhou, 2020 [124]
HA-DMSN (HA: hydroxyapatite	-	6.4	-	220	BMSCs cells; Rats	Bone tissue regeneration	HA-DMSN increased ALP activity, bone regeneration, and calcium deposits in vitro and in vivo, resulting in enhancing bone regeneration in the cranial bone defect model.	Lei, 2020 [125]
MSN_miR-26a@PEI–KALA	-	-	-	~109	SD rBMSCs	Bone loss	MSNs were able to guarantee the stability of RNA by protecting miR-26a from degradation. The formulation significantly increased osteogenesis with relatively small doses.	Yan, 2020 [126]
Ciprofloxacin-loaded Chitosan-MSNs	-	2.61	0.923	100 ± 13	-	Bone regeneration	MSNs provided slow release of the antibacterial drug for 9 h compared to 2 h without MSNs, thus suggesting better treatment for bone regeneration by delivering antibacterial drugs.	Hezma, 2020 [127]

## 6. Application of MSNs in Disease Diagnosis

**Figure 8:**
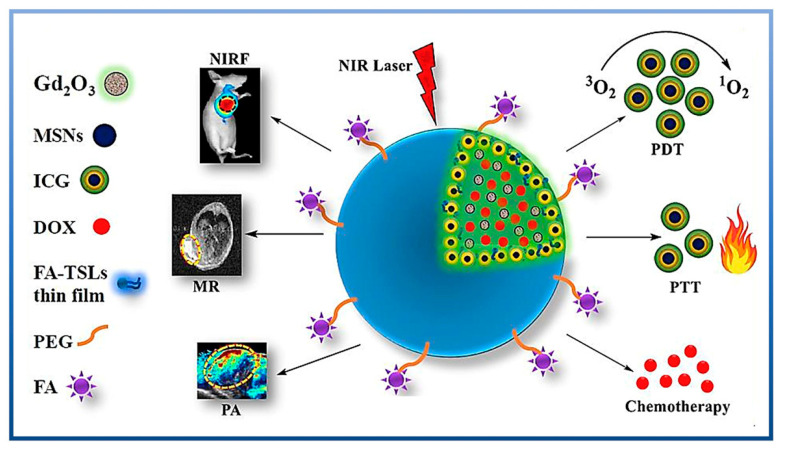
Application of MSNs-based nanoplatforms for cancer diagnosis and therapy. Adapted with permission from M. Abbasi et al. (2021) [128]. Copyright 2021 Elsevier.

Using nanoparticles as part of diagnosis or treatment of diseases such as cancer is one of the key approaches to improve biocompatibility or safety. Organic metals, polymers, and inorganic materials, especially silica, are the most frequently used materials for formulations. Thus far, many studies have reported that MSN-based nanoparticles can be used to detect cancer in diagnostic methods, such as magnetic resonance imaging, fluorescence imaging, ultrasound imaging, positron emission tomography (PET), photoacoustic imaging, or computed tomography (CT) [128] (Figure 8). Ovarian cancer, as one of the deadliest diseases in women, also requires more accurate and advanced detection to help patients receive early therapy to lower mortality and increase the survival rate. H. Liu et al. [129] used biotin-enriched dendritic MSNs with multiplex lateral flow immunoassay (LFIA) and the results showed MSNs’ capability to detect ovarian cancer biomarkers (CA125 and HE4). The results of this study demonstrated that MSNs have a high correlation coefficient of over 98%, which is higher than afforded by the commercial electrochemiluminescence method.

MSNs have also been studied for their characteristic properties, including their shape, as these properties can enhance their effectiveness as drug carriers. Rod-shaped mesoporous nanoparticles have demonstrated their usefulness for diagnostic imaging in cancer therapy using three-dimensional tomography [58]. In a study by M. Todea et al. [130], as a result of measuring Fe^3+^ EPR (electron paramagnetic resonance) parameters, monodispersed silica-based microspheres formed using alumina and iron oxide on a mesoporous silica core (AlFe@SiO_2_) showed considerably improved superparamagnetic behavior. Experiments of these particles in the body fluid revealed good stability and bioinert behavior of AlFe@SiO_2_, suggesting that they are a good platform in MRI imaging and hyperthermia for cancer treatment and diagnosis.

Recently, virus infection has been focused on by many studies seeking to improve efficiency and increase the accuracy of the reliable detection method. For instance, B.G. Tuna et al. [131] successfully fabricated MSNs based on fluorescein and their NSP12 gene-based diagnostic system demonstrated a strong signal for detection of SARS-Coronavirus-2. In addition, the detection process has several advantages, such as higher sensitivity in detecting the correct presence of virus in samples as compared to qRT-PCR and a faster detection time than the marketed product for detection of SARS-Coronavirus-2.

## 7. Clinical Studies of Silica-Nanoparticle-Based Systems

Today, nanomedicines are gaining attention for their ability to successfully deliver drugs in clinical studies. The effectiveness of nanomedicines—including inorganic nanoparticles—has also been demonstrated in commercial nanomedicines. Several studies demonstrated that nanomedicines can be used to treat cancer or various diseases (i.e., diabetes, cardiovascular disease) by reducing adverse effects of conventional therapy or by enhancing targeting efficiency to deliver drugs to specific sites; however, using nanomedicines still has some challenges, such as reproducibility and proper characterization of the systems [132,133]. Compared to other organic or inorganic nanoparticle types, silica nanoparticles have unique advantages; therefore, two clinical studies and eleven clinical trials have been conducted to ensure that the safety profile for human subjects is adequate [134] (Table 3). A clinical study using silica–gold nanoparticles (the NANOM-FIM trial) with 180 patients showed good reduction in coronary atherosclerosis along with acceptable levels of safety for use in humans. Compared to the XIENCE V stent, long-term outcomes and safety with this device showed high safety and a low mortality rate, as well as no major adverse cardiovascular events [135,136]. Another study showed that silica–lipid hybrid spheres (SLHs) can effectively increase the bioavailability of simvastatin in a phase I trial on healthy men. The formulation was well-tolerated in patients, without significant side effects [137]. In another clinical trial by A. Tan et al. [138], a silica nanoparticle–lipid hybrid formulation of ibuprofen (Lipoceramic-IBU) was tested in 16 healthy men. Herein, the silica nanoparticles were used to enhance the solubility and bioavailability of the poorly water-soluble drug. A comparison study with Nurofen^®^, a commercial product, revealed that the silica nanoparticle system (Lipoceramic-IBU) had enhanced bioavailability, with a 1.5-fold higher maximum plasma concentration. Furthermore, in a clinical study by K. Bukara et al. [139], the ordered mesoporous silica (OMS) was applied to improve solubilization of fenofibrate, a poorly water-soluble drug, resulting in higher absorption rates and extents with formulation fenofibrate-OMS as compared to a marketed product, Lipanthyl^®^. K. Bukara et al. also reported that single-dose administration of fenofibrate-OMS was well-tolerated by 12 Caucasian males.

According to a clinical study by E. Phillips et al. [140], a device using core-shell hybrid fluorescent silica nanoparticle tracers (^124^I-cRGDY–PEG–C dots) was well-tolerated in patients with metastatic melanoma. A reproducible pharmacokinetic signature was also observed for renal excretion in preclinical and clinical studies. This ultrasmall inorganic optical-PET imaging nanoparticle system showed neither toxicity nor adverse effects in clinical studies, suggesting that the device is safe for use in human disease diagnosis. A nonrandomized clinical trial by D.K. Zanoni et al. [141] applied an ultrasmall core-shell silica nanoparticle (Cornell prime dots) in fluorescence-guided sentinel lymph node (SLN) biopsy for 24 patients aged 18 years and older with head and neck melanoma. The results revealed the safety of the system, suggesting that the system can be a promising tool for enhancing mapping and SLN biopsy procedures while eliminating exposure to harmful consequences. Furthermore, a recent study by A.R. Rastinehad et al. [142] showed photothermal ablation of low- or intermediate-grade tumors in the prostate by a laser-excited gold–silica nanoshell (GSN) with a silica core and gold shell. Based on their safety study, these Auroshell-type particles were found to not cause any deleterious changes in genitourinary function or other serious complications. This study demonstrated that the approach using GSNs (Auroshell) could be a feasible treatment for patients with low- or intermediate-risk prostate cancers.

## 8. Biocompatibility, Toxicity, and Safety Issues of MSNs for Biomedical Applications

DDS using nanoparticles has been developed to increase the effectiveness of drugs to achieve the maximum therapeutic effect. However, along with improving formulation designs, safety-related aspects should not be overlooked. Modification of the physicochemical properties of nanoparticles can be the basis for controlling the level of toxicity, stability, and effectiveness of formulations and interaction between particle formulations and molecular targets in the body [143]. In present-day development of formulations, MSNs have received much attention for their usefulness in a variety of applications, including cancer therapy, tissue engineering, bioimaging, and theranostic purposes. On the other hand, several issues, such as drug release patterns, size of nanoparticles, and other concerns, such toxicity or biocompatibility, have emerged; therefore, to minimize adverse effects and maximize the effectiveness of MSNs-based DDS, in vitro and in vivo studies on those issues should be conducted before applying MSNs in clinical practice [23]. Studies about particle size have revealed that MSNs’ biodistribution might be affected by particle size; MSNs with very small diameters (5–6 nm) tend to suffer from fast clearance by the kidney. The shape of the nanoparticles was also found to affect the flow of distribution and duration of drug action in the body. Biodegradation and clearance of silica nanoparticles in organs can be managed by particle size, pore size, morphology, and surface chemistry. Generally, silica nanoparticles without functionalization are degraded via fast hydrolysis into ‘water-soluble silicic acid’ in the body [144]. Silicic acid is excreted via urine and hepatobiliary secretion and partly absorbed in the body [145], which clears concerns of bioaccumulation of silica nanoparticles. Researchers can consider incorporating either redox- or enzymatically cleavable constituents or hydrolytically stable components into silica nanoparticles during synthesis to achieve the targeted drug delivery or required clearance kinetics in organs where particles are distributed [146]. By using synthetic strategies, degradation and clearance kinetics of silica nanoparticles can be tunable to allow more safe and efficient applications in the body.

In previous research on silica nanoparticles, the results of a toxicity test experiment with a micropillar/microwell chip platform revealed that, when HepG2 cells were cultured in a 2D culture system in SF medium, the smaller nanoparticle size yielded a greater toxic effect with the decreased cell viability [147]. Moreover, toxicity of silica nanoparticles on HepG2 was found to be varied depending on culture medium or matrices in 3D scaffolds (i.e., non-toxic effects in the 3D culture using Matrigel). Based on this study, in vitro nanotoxicity results could be different among experimental settings, Thus, besides in vitro studies, it is suggested that toxicity profiles should be investigated in animal studies as well. In a review, J.G. Croissant et al. [148] overviewed several in vitro and in vivo studies about toxicity issues associated with silica nanoparticles and fully discussed that chemical constituents and structures of various types of silica nanoparticles, including colloidal silica nanoparticles, Stöber nanoparticles, or MSNs, can influence degrees and types of toxicity in tissues and alterations in immunity. For example, some previous studies demonstrated dependence of hemolysis on silanol concentration of silica nanoparticles [149]. Those studies suggest that non-covalent interaction between silanol groups in silica nanoparticles and phospholipids in blood cells could perturb cell membrane, causing hemolysis. In the case of MSNs, the porous structure could reduce the contact area between the particle and cell membrane and also effective silanol concentration, resulting in reduced hemolytic activity compared with colloidal or Stöber silica nanoparticles [149,150].

In toxicity-related in vivo studies carried out by Y.-D. Deng et al. [151], MSNs increased ROS and NLRP3 levels, and a significant change in the intestinal flora of mice was observed. In addition, MSNs also induced apoptosis-related protein caspase-3 and triggered damage to mitochondrial structures. According to a review by L. Chen et al. [152], some toxic effects in tissues after administration of silica nanoparticles were found to be related with specific mechanisms, including (1) proinflammatory responses, such as IL-6, IL-1β, TNF-α cytokines, or NLRP3 inflammasome activation; (2) oxidative stress by ROS generation via NADPH oxidase and MAPK (mitogen-activated protein kinase) signaling pathway; (3) autophagy dysfunction, etc. Further research provided evidence that silica nanoparticles might affect the immunity of tissues, hemocytes, and also major organs, such as the liver, spleen, lung, and heart. In several studies, silica nanoparticles showed size- or dose-dependent toxic effects on immune cells, such as dendritic cells, macrophages, or lymphocytes. Immunotoxic effects included genotoxicity, cytotoxicity, and dysfunction of immune cells, which should be investigated for different types of silica nanoparticles, including MSNs with various physicochemical properties. A.M. Mahmoud et al.’s [153] study showed that MSNs significantly induced dose-dependent damage in liver and kidney in rats via MSNs-triggered oxidative stress, inflammatory signals, and fibrosis. Furthermore, in in vivo studies by J. Li et al. [154], MSNs were found to affect hepatic metabolism after intravenous (IV) administration at a concentration of 20 mg/kg/day or after oral administration at 200 mg/kg/day for 10 days. Further pathological examination after IV administration of MSNs revealed induction of hepatic injury with increasing levels of AST/ALT and inflammatory factors, such as IL-1β, IL-6, and TNF-α. Based on proteomic and transcriptomic analyses, increases in GPX, SOD3, G6PD, HK, and PFK after IV administration of MSNs suggested elevation in glycolysis and pentose phosphate pathways, glutathione synthesis, as well as reduction in oxidative phosphorylation, TCA, and mitochondrial energy metabolism. Meanwhile, in this study, milder toxicity was expressed by oral delivery even at ten-fold higher doses than at doses of intravenous injection. These data can be used as a basis for further research on the subacute to long-term toxicity of MSNs in future therapy applications.

However, other in vivo studies showed that either changes in the cell cycle or toxicity induction through ROS/RNS (reactive nitrogen species) mediation were not caused by even bare MSNs without surface modifications [155]. Compared to amorphous non-porous silica nanoparticles (i.e., Stöber silica nanoparticles) with evidence of toxicities, including cell cycle changes or apoptosis, MSNs have been reported to be less toxic and more improved in toxicity profiles with further surface modifications. A comparison of bare MSNs and functionalized MSNs using HA and PEG in experiments on MDA-MB-231 and MCF10A cell lines revealed that both MSNs have safety, which can be the basis of their future biomedical applications [155]. Furthermore, another study on modification of MSNs described that, compared to non-functionalized MSNs, L-tartaric-acid-modified chiral MSNs had better in vitro and in vivo behaviors, including biodegradation, bio-adhesion, wettability, biocompatibility, and good blood compatibility, which suppress toxic effects [156]. In addition, the previous report mentioned that, although MSNs have larger surface areas as compared to non-porous silica nanoparticles of equivalent size, in general, due to porosity, MSNs still show lower toxicity from the point of view of hemolytic activity. Ultimately, further aspects regarding the parameters that can affect cytotoxicity of MSNs except for cell types or viability assays include particle size/surface charge, shape, dose, and functionalization [157]. Controlling physicochemical characteristics as well as choosing the appropriate dosage regimen (i.e., dose, dosing interval, and route of DDS administration) can diminish possible toxicities related with MSNs and overcome limitations of using MSNs.

## 9. Conclusions and Future Perspectives

Effective and controlled drug delivery has been an important issue in the biomedical field because it is closely related to therapeutic efficacy and low side effects after drug administration. With advanced nanotechnologies (i.e., polymers or lipid-based platforms), many different types of nanoplatforms were devised and tested in pharmaceutical research for treatment of cancer or other diseases. However, it remains challenging to achieve better therapeutic efficacy with conventional nanoplatforms as they have limitations to overcome physiological barriers. In this aspect, mesoporous silica nanoparticles (MSNs) have pharmaceutical advantages to be developed as drug carriers to load drug molecules at high concentrations and deliver drugs to the target sites in the body in a responsive manner to physiological stimuli. As described in this review, many reports evidenced that MSNs could be easily functionalized by enabling attachment of targeting moieties or responsive components to interact with cells/tissues in the physiological environment. Those particles could improve therapeutic platforms with pharmaceutically desirable properties of high surface area, manipulatable surface chemistry, biocompatibility, and physical stability with loads of cargo. As mentioned in the review, numerous effective drug delivery systems for disease treatment or tissue engineering could be generated based on the nanoplatform of MSNs due to their pharmaceutical properties.

Preclinical or clinical research with silica nanoparticles revealed that MSNs have many beneficial effects as drug carriers in DDS, which are helpful in treatment of various diseases. Although there are several clinical studies on silica nanoparticles, there are still considerations for commercial application or marketed drugs. In addition, several studies noted occurrence of toxicity and side effects when using silica nanoparticles. Moreover, in the context of morphology and modification of MSNs, surface chemistry, diameter size, pore size, and shape are also considered to determine the best formula with minimum toxicity and maximum efficacy. It is necessary to strengthen the in vitro and in vivo studies for investigating acute to long-term toxicity, immunity, or biodistribution issues so that the MSN-based formulations can then be evaluated in the clinical stage. As for design and formulation of MSNs applicable to clinical use, further thorough research and analysis should be conducted to determine therapeutic effects and any adverse responses possibly associated with medicines based on silica nanoparticles. Thus far, along with many nano-formulations and designs that have previously been studied, many reports have evidenced that MSNs-based DDS can provide a better therapeutic effect than free drugs. Changes in surface chemistry, control of diameter size, and high drug-loading capabilities can increase functionality of MSNs as drug carriers for various therapies. With various considerations of the MSNs formulation after thorough investigations of toxicity and biocompatibility, it can be hoped that, in the future, there will be a drug delivery innovation capable of increasing the therapeutic effect by minimizing potential side effects and improving clinical outcomes. In addition to advances in methods of generating various types of functionalized MSNs, optimization of the technologies should be accomplished for further pharmaceutical applications. Progress in synthesis procedures, physicochemical characterization, and scale-up production of MSN-based nanoplatforms would enable MSNs to be applied as pharmaceuticals in a wide range of biomedical fields.

## Figures and Tables

**Table 1:** Various mechanisms through which stimuli-responsive controlled drug delivery systems of MSNs work [25].

Stimuli	Mechanism for Cancer Treatment	Research Reference
pH	Extracellular pH of malignant tissues and inflammatory tissues are in slightly acidic condition, i.e., around 6.0–7.0, whereas pH in normal healthy tissues is 7.4. Therefore, once reaching the target sites, MSNs are triggered, ensuring the release of the drug at the therapeutic concentration.	Sia et al., 2022 [76]
Redox state	Reduced Glutathione (GSH) is overexpressed in neoplastic tissue compared to healthy tissues. Therefore, under the different microenvironments of GSH levels in neoplastic and healthy tissues, disulfide cleavage of GSH-sensitive MSNs can be triggered to promote drug or gene delivery in tumor sites.	Hu et al., 2018 [77]
Temperature	Temperature is slightly increased by 4–5 °C in cancer. Based on this mechanism, a temperature-responsive controlled release system can be developed by grafting a temperature-sensitive nano-switch on the surface of MSNs and MSNs can release drugs with increased penetrability, specifically in cancer tissues. As for the temperature-sensitive component, polymers based on poly-N-isopropylacrylamide (PNIPAM) and its derivatives can be used.	Wei et al., 2009 [78]
Enzyme	Employing upregulated enzymes in any pathological condition can be used as a triggering point for designing stimuli-responsive MSNs that catalyze any chemical reactions in cancer tissues. For instance, matrix metalloproteinase in the cancer microenvironment or phospholipases in pancreatic cancer, etc., can be used in tailoring MSNs by changing linkers and capping agents on their functionalized surface.	Zou et al., 2015 [79]
Light	By incorporating a light-triggering system in MSNs, photodynamic or photothermal (PTT) therapy can be feasible by releasing drugs in tumor sites. For example, fluorophores such as near-infrared (NIR) dyes can be used for preparing MSNs for bioimaging or PTT.	Yang et al., 2012 [80]
Magnetic stimuli	Magnetic nanoparticles embedded in MSNs can be efficiently used in cancer therapy by using the activity of magnetic hyperthermia. The heat generated by magnetic nanoparticles under application of alternating magnetic field can facilitate efficiency of cancer treatment.	Knežević et al., 2013 [81]
Ultrasound	MSNs-based nanocomposites can be used for ultrasound-triggered drug delivery to cancer sites. Ultrasound and MSNs showed synergistic effects for cancer treatment and also could be applied for photoacoustic-imaging-guided chemotherapy.	Paris et al., 2018 [82]

**Table 3:** Study on a clinical trial on silica nanoparticles (ClinicalTrials.gov (accessed on 2 February 2023)) [134].

Year (Actual Study Start)	Condition	Recruitment Status	Location	Identifier (Website Accessed on 2 February 2023)
2007	Stable angina Heart failure Atherosclerosis Multivessel coronary artery disease	Completed	Netherlands Russian Federation	NCT01270139 (NANOM-FIM) https://clinicaltrials.gov/ct2/show/NCT01270139
2008	Head and neck cancer	Completed	United States, Arizona United States, Texas	NCT00848042 (Auroshell) https://clinicaltrials.gov/ct2/show/NCT00848042
2010	Coronary artery disease Atherosclerosis	Terminated	Netherlands Russian Federation	NCT01436123 (NANOM-PCI) https://clinicaltrials.gov/ct2/show/NCT01436123
2011	Newly diagnosed or recurrent metastatic melanoma patients Malignant brain tumors	Active, not recruiting	United States, New York	NCT01266096 https://clinicaltrials.gov/ct2/show/NCT01266096
2014	Head and neck melanoma	Recruiting	United States, New York	NCT02106598 https://clinicaltrials.gov/ct2/show/NCT02106598
2016	Neoplasms of the prostate	Completed	United States, Maryland United States, Michigan United States, New York United States, Texas	NCT02680535 https://clinicaltrials.gov/ct2/show/NCT02680535
2018	Brain cancer Pituitary adenoma	Active, not recruiting	United States, New York	NCT03465618 https://clinicaltrials.gov/ct2/show/NCT03465618
2021	Prostate cancer	Recruiting	United States, New York	NCT04167969 https://clinicaltrials.gov/ct2/show/NCT04167969

## Data Availability

Not applicable.
